# Glucomannan promotes *Bacteroides ovatus* to improve intestinal barrier function and ameliorate insulin resistance

**DOI:** 10.1002/imt2.163

**Published:** 2024-01-03

**Authors:** Qixing Nie, Yonggan Sun, Wenbing Hu, Chunhua Chen, Qiongni Lin, Shaoping Nie

**Affiliations:** ^1^ State Key Laboratory of Food Science and Resources, China‐Canada Joint Lab of Food Science and Technology, Key Laboratory of Bioactive Polysaccharides of Jiangxi Province Nanchang University Nanchang China; ^2^ College of Grain Science and Technology Jiangsu University of Science and Technology Zhenjiang China

**Keywords:** aryl hydrocarbon receptor, *B. ovatus*, glucomannan, indoleacetic acid

## Abstract

Bioactive dietary fiber has been proven to confer numerous health benefits against metabolic diseases based on the modification of gut microbiota. The metabolic protective effects of glucomannan have been previously reported in animal experiments and clinical trials. However, critical microbial signaling metabolites and the host targets associated with the metabolic benefits of glucomannan remain elusive. The results of this study revealed that glucomannan supplementation alleviated high‐fat diet (HFD)‐induced insulin resistance in mice and that its beneficial effects were dependent on the gut microbiota. Administration of glucomannan to mice promoted the growth of *Bacteroides ovatus*. Moreover, colonization with *B. ovatus* in HFD‐fed mice resulted in a decrease in insulin resistance, accompanied by improved intestinal barrier integrity and reduced systemic inflammation. Furthermore, *B. ovatus*‐derived indoleacetic acid (IAA) was established as a key bioactive metabolite that fortifies intestinal barrier function via activation of intestinal aryl hydrocarbon receptor (AhR), leading to an amelioration in insulin resistance. Thus, we conclude that glucomannan acts through the *B. ovatus*‐IAA‐intestinal AhR axis to relieve insulin resistance.

## Highlights

- Supplementation with glucomannan alleviates insulin resistance.
- Glucomannan promotes the growth of *Bacteroides ovatus*, accompanied by improved intestinal barrier integrity and reduced systemic inflammation.
- *B. ovatus*‐derived indoleacetic acid is a key bioactive metabolite that fortifies intestinal barrier function via the activation of intestinal aryl hydrocarbon receptor.

## Graphical abstract

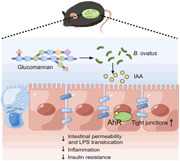

## INTRODUCTION

Insulin resistance is defined as a decreased insulin‐mediated control of glucose metabolism in host tissues [1]. It is caused by a combination of genetic and environmental factors. Accumulating evidence suggests that dysbiosis of gut microbiota is involved in the onset and progression of obesity and obesity‐related complications, such as insulin resistance and type 2 diabetes (T2D) [2, 3]. Gut microbiota and their metabolites can impact host insulin resistance via multiple pathways. For instance, the gut microbiota of T2D patients has an increased branched‐chain amino acid (BCAA) biosynthesis potential, among which *Segatella copri* and *Phocaeicola vulgatus* are the dominant species driving the biosynthesis of BCAAs, eventually leading to insulin resistance [4, 5]. The increased level of bacteria‐derived imidazole propionate also reportedly contributes to insulin resistance by activating the p38γ‐mTOR1‐S6K1 signaling pathway [6]. On the contrary, supplementation with *Akkermansia muciniphila* increases mucus layer thickness and fortifies intestinal barrier function, which reverses high‐fat diet (HFD)‐induced metabolic endotoxemia and insulin resistance [7, 8]. Furthermore, *Bacteroides uniformis* and *Bacteroides acidifaciens* intervention also prevents obesity and improves insulin sensitivity in mice, which might be associated with regulation of intestinal immunity and homeostasis [9, 10]. Importantly, gut microbiota dysbiosis is associated with disrupted intestinal barrier function and metabolic endotoxemia, which is a nonnegligible factor of low‐grade inflammation in metabolic diseases [11]. Consequently, strategies to modify gut microbiota and enhance intestinal barrier function have been proposed to prevent insulin resistance and related metabolic diseases.

Experimental and clinical studies have established that direct targeting and remodeling of the gut microbiota is an effective strategy to prevent and alleviate metabolic diseases. For instance, supplementation with dietary fiber could modulate the gut microbiota to improve intestinal barrier function, alleviating insulin resistance by reducing metabolic endotoxemia and chronic inflammation [11]. Glucomannan is a popular bioactive dietary fiber derived from several herbs, such as Konjac and *Dendrobium officinale*. Among them, konjac glucomannan (KGM) is one of the most extensively studied glucomannan, characterized by β‐(1 → 4)‐linked d‐glucosyl and d‐mannosyl residues as the main chain with branches through β‐(1 → 6)‐glucosyl units [12]. In many European countries, KGM is being widely marketed for the management of overweight or obesity. In a placebo‐controlled, diet‐controlled trial, supplementation with KGM promoted bowel movement and improved colonic ecology (increase abundance of bifidobacteria and lactobacilli and levels of fecal SCFAs) in constipated adults [13]. In addition, KGM intervention also ameliorates glycemic control, blood lipid profile, systolic blood pressure in high‐risk diabetic individuals [14]. Our previous studies noted that the administration of bioactive dietary fiber (including KGM) could improve glucose homeostasis and exert anti‐inflammatory effects by enhancing the growth of specific antiobesogenic gut bacteria and the production of bacteria‐derived metabolites [15, 16]. Furthermore, KGM was previously reported to improve obesity and insulin resistance in HFD‐fed mice [17, 18], whereas its detailed molecular mechanism on insulin resistance remains to be elucidated.

Dietary fiber escapes gastrointestinal digestion and is degraded by the gut microbiota into nutrients that are tractable to the host and affect the composition of microbes. We hypothesize KGM could ameliorate insulin resistance by regulating gut microbiota and related metabolites. In the present study, we investigate the molecular mechanisms of KGM on insulin resistance by physiology, metabolomics, and genomics analysis, and demonstrated glucomannan promotes *Bacteroides ovatus* to improve intestinal barrier function and ameliorate insulin resistance. Our results shed light on the mechanism by which glucomannan alleviates insulin resistance via gut microbiota, thereby offering new insights into the amelioration of insulin resistance by bioactive dietary fiber.

## RESULTS

### KGM alleviates HFD‐induced insulin resistance

**Figure 1:**
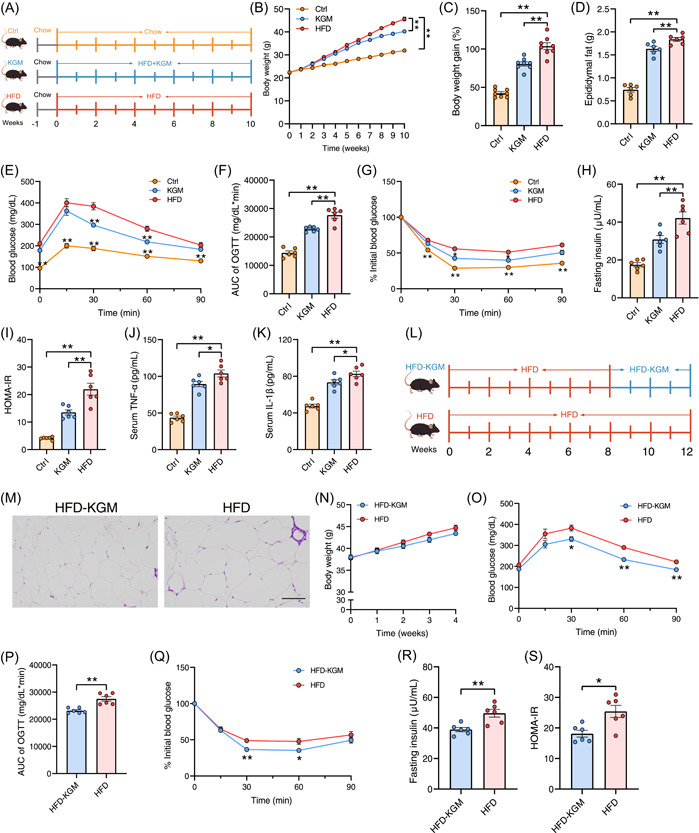
Konjac glucomannan (KGM) ameliorates high‐fat diet (HFD)‐induced insulin resistance and metabolic disorders. (A) Experimental scheme for (B–K), *n* = 6 mice/group. Chow‐fed and HFD‐fed mice were treated with phosphate‐buffered solution (PBS) (Ctrl and HFD groups) and KGM (200 mg/kg, KGM group) for 10 weeks by oral gavage. (B) Body weight. (C) Body weight gain. (D) Epididymal fat weight. (E, F) Oral glucose tolerance test (OGTT) of the three groups and the associated area under the curve (AUC) values. (G–I) Insulin tolerance test (ITT), fasting insulin level, and homeostasis model assessment of insulin resistance (HOMA‐IR) index following KGM treatment. (J, K) Changes in serum tumor necrosis factor‐α and interleukin‐1β levels after KGM treatment. (L) Experimental scheme for (M–S), *n* = 6 mice/group. Obese mice were treated with PBS (HFD group) and KGM (200 mg/kg, HFD‐KGM group) for 4 weeks by oral gavage. (M) Hematoxylin and eosin staining of epididymal fat from mice in the HFD or HFD‐KGM group. (N) Body weight. (O, P) OGTT of the two groups and the associated AUC values. (Q–S) ITT, fasting insulin level, and HOMA‐IR index after KGM treatment. All data are presented as means ± SEMs. In (B–K) *p < 0.05 and **p < 0.01 versus the HFD group. In (N–S) *p < 0.05 and **p < 0.01 versus the HFD group.

To investigate the metabolic protective effect of KGM, it was administrated to HFD‐induced insulin‐resistant mouse models (Figure 1A). As anticipated, body weight, epididymal fat weight, fasting insulin level, glucose intolerance, and HOMA‐IR were significantly increased in HFD‐fed mice compared to those on a chow diet (Figure 1B−I). In contrast, 10‐week treatment with KGM significantly decreased body weight and epididymal fat weight, compared to the HFD group (Figure 1B−D), with no significant difference in energy intake (Figure S1A). Supplementation with KGM also improved glucose tolerance and insulin resistance under HFD conditions, along with decreased serum tumor necrosis factor‐α (TNF‐α) and interleukin‐1β (IL‐1β) levels (Figure 1E−K). Meanwhile, the level of serum total triglyceride (TG), nonesterified fatty acid (NEFA), ALT, and AST were also significantly diminished following KGM administration, suggesting that KGM may contribute to the hypolipidemic and hepatoprotective effect in obese insulin‐resistant mice (Figure S1B–F). Then, the influence of KGM on obese mice was further investigated (Figure 1L). Mice were treated with 8 weeks of HFD and then orally gavaged with KGM for 4 more weeks. KGM ameliorated glucose intolerance and insulin resistance with no significant effect on energy intake and body weight (Figures 1N−S and S1G). More importantly, the size of adipocytes in the white adipose tissue, as well as serum TG and ALT levels, were decreased following KGM treatment (Figures 1M and S1H−K).

### KGM improves intestinal barrier function and metabolic endotoxemia

**Figure 2:**
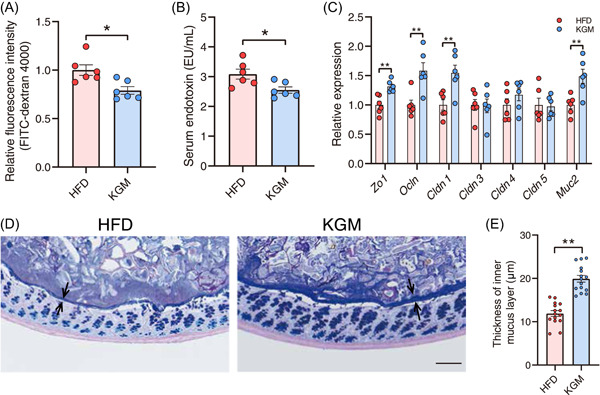
Konjac glucomannan (KGM) improves intestinal barrier function. (A) Intestinal permeability measured by plasma fluorescence intensity after fluorescein isothiocyanate‐dextran 4000 gavage. (B) Serum endotoxin level. (C) The relative messenger RNA expression level of *Zo1*, *Ocln*, *Cldn1*, *Cldn3*, *Cldn4, Cldn5*, and *Muc2* in colonic tissue after KGM treatment. (D) Carnoy‐fixed colonic tissue sections were stained with Alcian blue/periodic acid‐Schiff. Scale bars, 100 µm. (E) Blinded colonic mucus layer measurements from Alcian blue‐stained sections. All data are presented as means ± SEMs. In (A–E) *p < 0.05 and **p < 0.01 versus the HFD group. FITC, fluorescein isothiocyanate; HFD, high‐fat diet.

Intestinal barrier dysfunction and low‐grade inflammation are distinctive features of metabolic diseases. KGM administration significantly reduced intestinal permeability (Figure 2A) and serum endotoxin levels (Figure 2B) compared with HFD‐fed control mice. Following this, the expression of genes related to barrier function was examined. The results were consistent with the aforementioned intestinal barrier function, with KGM upregulating the expression of intestinal *Zo1*, *Ocln*, *Cldn1*, and *Muc2* (Figure 2C). The mucus layer is a dynamic barrier that is constantly replenished through the secretory activity of goblet cells. In the current study, KGM increased the thickness of the mucus layer, as determined by Alcian blue staining (Figure 2D−E). Collectively, these results signal that KGM could improve intestinal barrier function in obese mice.

### KGM influences microbiota composition and increases the abundance of *B. ovatus*

**Figure 3:**
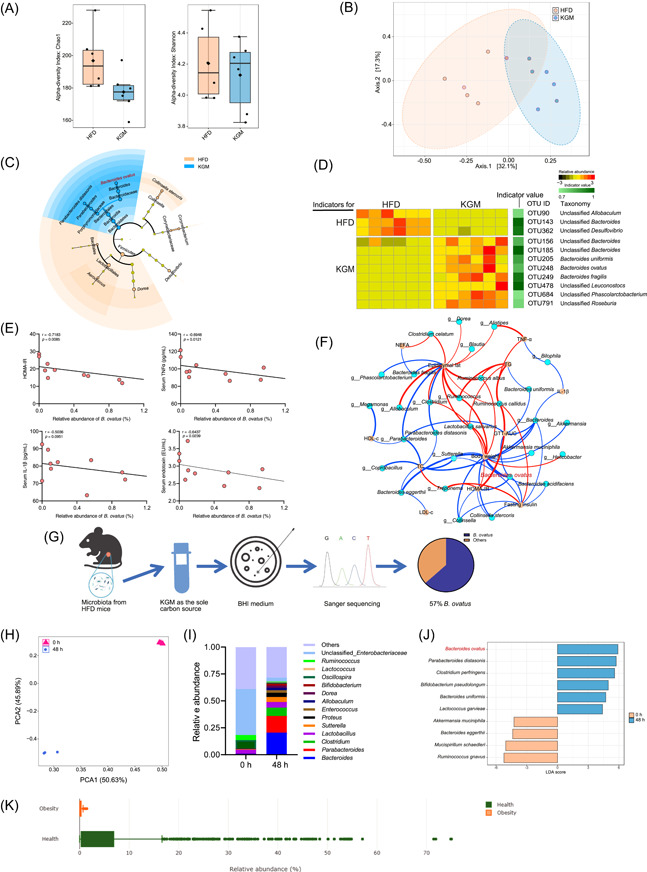
Konjac glucomannan (KGM) alters microbiota composition and increases *Bacteroides ovatus* abundance in mice with insulin resistance. (A) α‐diversity of the gut microbiota between the high‐fat diet (HFD) and KGM groups, as indicated by the Chao1, and Shannon indices. (B) Partial least squares discriminant analysis (PLS‐DA) using the Bray–Curtis distance. (C) A comparison of the relative abundance of bacteria between the HFD and KGM groups was performed by linear discriminant analysis effect size (LEfSe). (D) The Operational Taxonomic Units (OTUs) associated with each group were identified by indicator analysis. The heat map depicts the relative abundance and indicator values of indicator OTUs with a cutoff of adjusted *p* < 0.05 and indicator value > 0.7. (E) Correlative analysis of *B. ovatus* abundance with homeostasis model assessment of insulin resistance index, serum tumor necrosis factor‐α, interleukin‐1β, and endotoxin levels. Correlations between variables were assessed by linear regression analysis. Linear correction index *R* and *P* values were calculated. (F) Correlation network of insulin resistance‐related parameters and gut microbiota; the edges are colored based on the positive or negative Spearman correlation coefficients, with red indicating a positive correlation and blue indicating a negative correlation, and the stronger the correlation, the thicker the edge. (G) Schematic diagram of in vitro fermentation. (H) Composition of gut microbiota after in vitro fermentation with KGM. (I) Relative abundance at genus level after in vitro fermentation. (J) A comparison of the relative abundance of bacteria between the 0 and 48 h groups was made by LEfSe. (K) Relative abundance of *B. ovatus* in obese individuals compared with healthy individuals (mBodyMap).

Dietary fiber cannot be digested and absorbed by the host and passes through the upper gastrointestinal tract, and is fermented by gut bacteria, selectively stimulating the growth and activity of specific bacteria. To investigate the changes in gut microbiota induced by KGM administration, the microbiota of colonic content was analyzed by 16S ribosomal RNA (rRNA) gene sequencing. The α diversity between the two groups was comparable, as indicated by Chao1, and Shannon indices (Figure 3A). At the same time, β diversity indicated that the composition of the microbiota in mice in the KGM group was significantly different from those in the HFD group (Figure 3B). At the phylum level, KGM increased the abundance of Bacteroidetes and decreased that of Firmicutes (Figure S2A). Analysis of microbiota composition and the linear discriminant analysis (LDA) effect size (LEfSe) method revealed that the KGM group was characterized by genus *Bacteroides* and *Parabacteroides*, whereas *Collinsella*, *Corynebacterium*, *Aerococcus*, *Dorea*, and *Desulfovibrio* were considered as dominant bacteria in the HFD group (Figure 3C). Specifically, *Bacteroides ovatus* and *Parabacteroides distasonis* were significantly increased after KGM treatment (Figures 3C and S2B). Thereafter, indicator species analysis was carried out to explore the specific alteration in bacterial abundance at the Operational Taxonomic Unit (OTU) level, and the results demonstrated an increased abundance of *B. ovatus* after KGM intervention (Figure 3D). More importantly, the abundance of *B. ovatus* was negatively correlated with HOMA‐IR, serum endotoxin level, and TNF‐α level (Figure 3E,F).

*Bacteroides* are regarded as generalists for the degradation of dietary fiber based on their broad glycan utilization capabilities. An in vitro screening experiment was designed to investigate KGM‐influenced bacteria. Briefly, a medium was prepared with KGM as the sole carbon source, which was subsequently incubated with fecal microbiota from mice with insulin resistance. Next, the fermented samples were serially diluted in sterile PBS and spread on BHI agar plates. Finally, monoclonal colonies were collected and analyzed using Sanger sequencing (Figure 3G). The analysis found that KGM promoted the growth of *B. ovatus*, with 57% of all colonies characterized as *B. ovatus* under in vitro fermentation. The composition of bacteria was also significantly altered by KGM fermentation, especially since the abundance of the *Bacteroides* and *Parabacteroides* was substantially increased (Figures 3G−I and S2C). In line with the above results from 16S rRNA gene sequencing in mice, KGM fermentation also increased the abundance of *B. ovatus* and *P. distasonis*, while *B. ovatus* could effectively degrade KGM to promote their growth (Figures 3J and S2D−E). Moreover, the relationship between the abundance of *B. ovatus* and insulin resistance was examined. Briefly, mBodyMap (a curated database for microbes across the human body and their associations with health and diseases) was employed to validate changes in the abundance of *B. ovatus* under different host statuses [19], and a significant reduction in *B. ovatus* abundance was observed in obese individuals (Figure 3K).

### Alleviation of insulin resistance by KGM is microbiota‐dependent

To investigate whether the KGM‐influenced microbiota directly benefited insulin resistance, intestinal commensal bacteria were removed with antibiotics in obese mice and then treated with KGM (Figure S3A). Body weight, energy intake, glucose tolerance, and HOMA‐IR were comparable between mice in the vehicle and KGM groups, inferring that the beneficial effect of KGM on insulin resistance is directly mediated by gut microbiota (Figure S3B–H). In addition to the aforestated experiment, fecal microbial transplantation (FMT) experiment was conducted, whereby microbiota from KGM or HFD donors were transplanted into antibiotic‐treated obese mice (Figure S3I). As expected, mice that received KGM microbiota (KGM‐FMT) exhibited improvements in glucose tolerance and insulin resistance, with no substantial influence on body weight and energy intake (Figure S3J–P). Taken together, these results implied that gut microbiota mediates the beneficial effects of KGM on insulin resistance.

### *B. ovatus* protects mice against insulin resistance and improves intestinal barrier function

**Figure 4:**
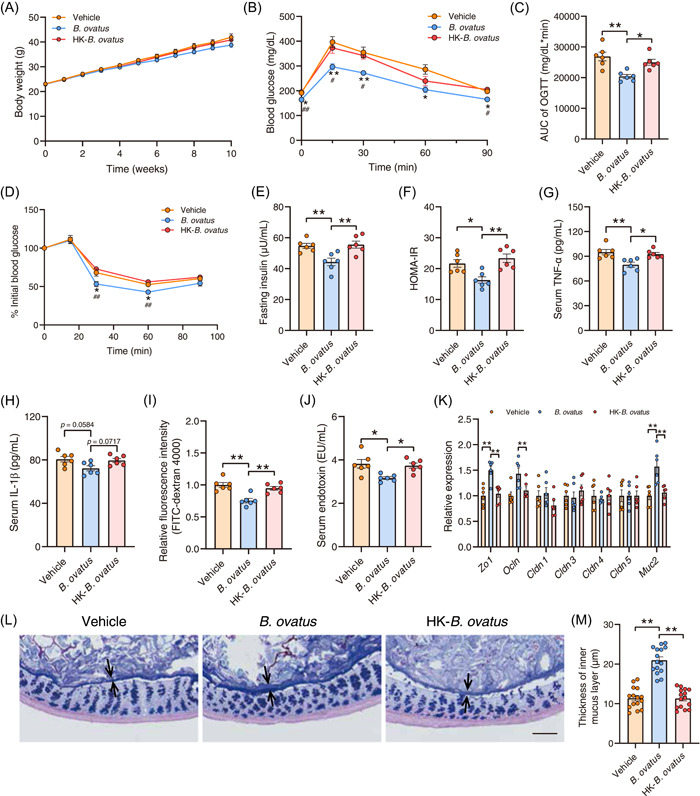
*Bacteroides ovatus* alleviates insulin resistance and improves intestinal barrier function. High‐fat diet (HFD)‐fed mice were treated with phosphate‐buffered solution (PBS) (Vehicle group), *B. ovatus* (*B. ovatus* group), or heat‐killed *B. ovatus* (HK‐*B. ovatus* group) strain once weekly for 10 weeks by oral gavage, *n* = 6 mice/group. (A) Body weight, (B, C) Oral glucose tolerance test of the three groups, and the associated area under the curve (AUC) values. (D–F) Insulin tolerance test, fasting insulin level, and homeostasis model assessment of insulin resistance index following *B. ovatus* treatment. (G, H) Changes in serum tumor necrosis factor‐α and interleukin‐1β levels after *B. ovatus* treatment. (I) Intestinal permeability was measured by plasma fluorescence intensity after fluorescein isothiocyanate‐dextran 4000 gavage. (J) Serum endotoxin level. (K) The relative messenger RNA expression level of *Zo1*, *Ocln*, *Cldn1*, *Cldn3*, *Cldn4*, *Cldn5*, and *Muc2* in colonic tissue after *B. ovatus* treatment. (L) Carnoy‐fixed colonic tissue sections were stained with Alcian blue/periodic acid‐Schiff. Scale bars, 100 µm. (M) Blinded colonic mucus layer measurements from Alcian blue‐stained sections. All data are presented as means ± SEMs. In (A–M) *p < 0.05 and **p < 0.01 versus the vehicle or *B. ovatus* group.

Given the findings from our in vitro and in vivo studies, the causality between *B. ovatus* and the alleviation of insulin resistance was further examined. Mice were fed with HFD and then administrated with either PBS, *B. ovatus*, or heat‐killed *B. ovatus*. Compared with the vehicle group, treatment with *B. ovatus* had no effects on the mice's body weight during the experiment, whereas it improved the impaired glucose metabolism induced by HFD (Figure 4A−F). *B. ovatus* significantly decreased levels of, fasting insulin, HOMA‐IR index, and serum TNF‐α. Moreover, glucose tolerance and insulin sensitivity were significantly enhanced by the bacterial intervention compared to control animals under HFD conditions, as evidenced by the OGTT and ITT results (Figure 4A−H). In contrast, metabolic protective effects were not observed in mice treated with heat‐killed *B. ovatus* (Figure 4A−H). Furthermore, gavage with living, but not heat‐killed, *B. ovatus* significantly decreased serum TG and AST level, with no influence on serum levels of TC, LDL‐c, HDL‐c, NEFA, and ALT (Figure S4A‐F). With the improvement in intestinal barrier function in mice treated with KGM, the influence of *B. ovatus* on intestinal permeability was further explored in HFD‐induced obese mice. *B. ovatus* effectively reduced intestinal permeability and level of serum endotoxin, as well as increased the gene expression level of intestinal *Zo1* and *Muc2* (Figure 4I−K). Simultaneously, HFD‐induced reduction of the thickness of the mucus layer was restored by *B. ovatus* intervention, demonstrating the protective effects of *B. ovatus* on intestinal barrier integrity (Figure 4L−M).

### *B. ovatus*‐derived indoleacetic acid (IAA) alleviates insulin resistance by activating AhR

**Figure 5:**
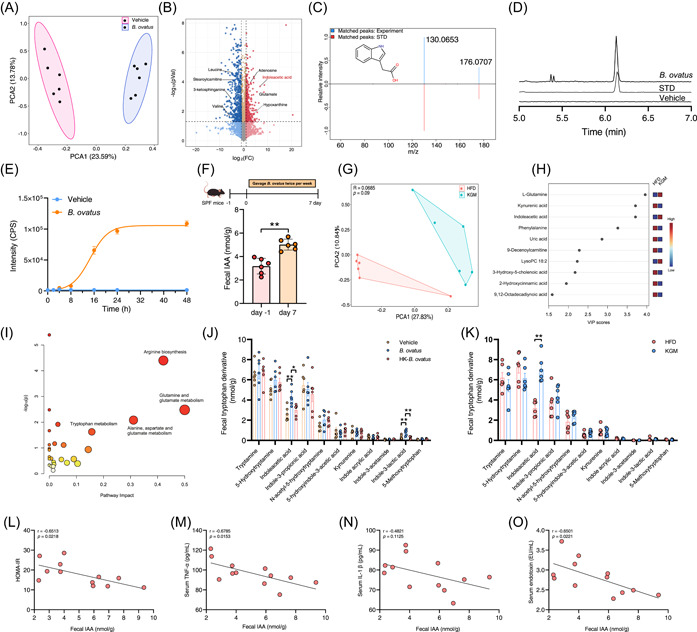
*Bacteroides ovatus* produces indoleacetic acid (IAA) and is negatively correlated with insulin resistance. (A) Nontargeted metabolomic profile of the supernatant of *B. ovatus* and GAM medium. (B) Volcano plot of metabolomic data in the vehicle and *B. ovatus* groups. (C) The MS/MS spectra of the IAA standard (bottom) and detected IAA from *B. ovatus*‐incubated samples (top). (D) Extracted ion chromatograms of IAA from cultured *B. ovatus* samples, medium control (vehicle), and IAA standard. (E) The production of IAA by *B. ovatus* with incubation time. (F) Mice were gavaged with *B. ovatus* strain twice per week for 1 week, and the feces were collected for IAA quantification. (G) Nontargeted metabolomic profile of fecal samples in the high‐fat diet and konjac glucomannan (KGM) groups. (H) Variable importance in projection score of KGM‐influenced fecal metabolites. (I) The enriched pathways were calculated from the mummichog algorithm based on the nontargeted metabolomics analysis of fecal samples following KGM intervention. (J, K) Changes in fecal tryptophan metabolites after KGM and *B. ovatus* treatment. (L–O) Correlative analysis of IAA level with homeostasis model assessment of insulin resistance index, serum tumor necrosis factor‐α, interleukin‐1β, and endotoxin levels. Correlations between variables were assessed by linear regression analysis. Linear correction index *R* and *P* values were calculated. (A–C and G–I is nontargeted metabolomics, D–F and J–K is targeted metabolomics).

The gut microbiota influences host metabolism via the generation of various metabolites that interact with the host as functional signaling molecules in multiple organs. Based on the significant differences in metabolic protective effect between the *B. ovatus* and heat‐killed *B. ovatus* groups, we speculated that this phenomenon may result from *B. ovatus*‐derived metabolites. Therefore, *B. ovatus* was cultured in a gifu anaerobic medium (GAM) medium for 48 h, and nontargeted metabolomics was conducted to identify *B. ovatus*‐derived metabolites in bacterial culture supernatants. The metabolic profile of the culture with *B. ovatus* significantly differed from that with the vehicle medium, insinuating that key metabolites derived from *B. ovatus* may contribute to the metabolic protective effects (Figure 5A). Nontargeted metabolomic profiling yielded 5984 features, and the subsequent volcano plot displayed that the level of IAA was significantly increased in *B. ovatus* culture supernatants, with the matched MS/MS fragmentation spectra in standard and experiment samples observed (Figure 5B,C). Additionally, a targeted liquid chromatography–tandem mass spectrometry (LC‐MS/MS) method was constructed to validate the production of IAA by *B. ovatus*. Indeed, *B. ovatus* significantly increased the concentration of IAA both in vitro and in vivo (Figure 5D‐F). Noteworthily, antibiotics treatment significantly decreased the level of fecal IAA, which was rescued by FMT (Figure S5A).

In parallel, nontargeted metabolomics was performed in the feces of the HFD and KGM groups. The results showed that KGM intervention altered the metabolic profile of gut microbiota, leading to increased levels of l‐glutamine, IAA, and LysoPC 18:2, along with decreased concentrations of kynurenic acid, phenylalanine, uric acid, 9‐decenoylcarnitine, 3‐hydroxy‐5‐cholenoic acid, 2‐hydroxycinnamic acid, and 9,12‐octadecadiynoic acid (Figure 5G,H). Analyses of possible pathways affected by KGM in mice were based on the influenced metabolites, and KGM intervention altered multiple metabolic pathways, especially arginine biosynthesis; glutamine and glutamate metabolism; alanine, aspartate and glutamate metabolism; as well as tryptophan metabolism (Figure 5I). To validated the observations from nontargeted metabolomics, the concentration of tryptophan metabolism‐related metabolites in feces was also quantified via targeted metabolomics, and the IAA level was increased following KGM and *B*. ovatus treatment, which was also negatively correlated with HOMA‐IR and the levels of serum endotoxin and TNF‐α (Figure 5J−O).

**Figure 6:**
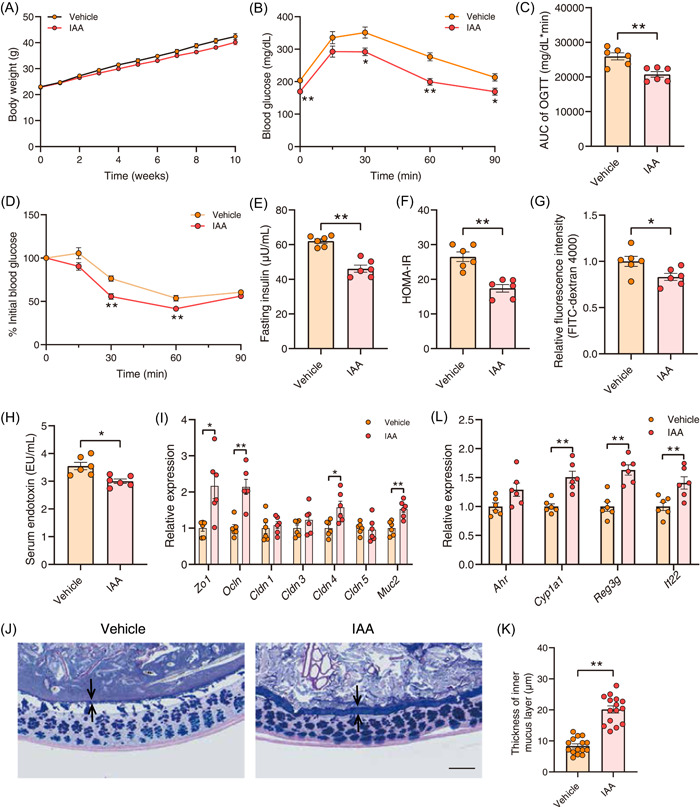
Indoleacetic acid (IAA) alleviates insulin resistance through AhR activation. High‐fat diet‐fed mice were treated with phosphate‐buffered solution (vehicle group) or IAA (IAA group) three times per week for 10 weeks by oral gavage, *n* = 6 mice/group. (A) Body weight. (B, C) Oral glucose tolerance test of the two groups and the associated area under the curve (AUC) values. (D–F) ITT, fasting insulin level, and homeostasis model assessment of insulin resistance index after IAA treatment. (G) Intestinal permeability was measured by plasma fluorescence intensity after fluorescein‐isothiocyanate‐dextran 4000 gavage. (H) Serum endotoxin level. (I) The relative messenger RNA expression level of *Zo1*, *Ocln*, *Cldn1*, *Cldn3*, *Cldn4*, *Cldn5*, and *Muc2* in colonic tissue after IAA treatment. (J) Carnoy‐fixed colonic tissue sections were stained with Alcian blue/periodic acid‐Schiff. Scale bars, 100 µm. (K) Blinded colonic mucus layer measurements from Alcian blue‐stained sections. (L) The relative expression level of intestinal *Ahr*, *Cyp1a1*, *Reg3g*, *Il22* after IAA treatment. All data are presented as means ± SEMs. In (A–L) *p < 0.05 and **p < 0.01 versus the vehicle group.

Based on the above‐mentioned findings, IAA might be a key metabolite that mediates the beneficial effects of KGM and *B. ovatus* on insulin resistance. To investigate their correlation, mice were fed with HFD and then administrated with either PBS or IAA for 10 weeks. Supplementation with IAA did not affect the body weight of mice but significantly improved glucose tolerance and insulin resistance compared with the vehicle group (Figure 6A−F). Simultaneously, IAA reduced intestinal permeability and level of serum endotoxin, and increased the gene expression level of intestinal *Zo1*, *Ocln*, *Cldn4*, and *Muc2*, as well as the thickness of the mucus layer (Figure 6G−K). Tryptophan is an essential amino acid for humans that is supplied by dietary protein. It can be metabolized by gut microbiota to produce indole derivatives, including indole‐3‐aldehyde, indoleacrylic acid, IAA, and so forth, which are ligands of the aryl hydrocarbon receptor (AhR). As expected, treatment with IAA significantly increased the relative gene expression level of *Cyp1a1*, *Reg3g*, and *Il22* (Figure 6L). Furthermore, the AhR signaling pathway was also activated after KGM and *B. ovatus* treatment (Figure S5B,C). Considering that short‐chain fatty acids (SCFAs) have been reported to activate AhR, the fecal SCFAs profile of mice treated with *B. ovatus* or KGM was examined, and the results revealed no significant changes in SCFAs among the different groups (Figure S5D,E). Collectively, our results demonstrated that the AhR signaling pathway, which is stimulated by *B. ovatus*‐derived IAA, plays a pivotal role in alleviating insulin resistance through KGM.

## DISCUSSION

The human gut microbiota plays a vital role in host immunomodulation, nutrient metabolism, and maintenance of structural integrity of the intestinal barrier. The intestinal barrier protects the host against physical, chemical, and microbial challenges in the gastrointestinal tract. Gut barrier integrity can be influenced by many intrinsic and extrinsic factors, including genetic predisposition, HFD diet, antibiotics, alcohol, circadian rhythm, psychological stress, and aging [20]. Chronic disruption of the intestinal barrier can facilitate the translocation of LPS into the circulation, a phenomenon commonly referred to as metabolic endotoxemia, which results in systemic, low‐grade inflammation. On the other hand, improvements in barrier function by prebiotics or probiotics led to reduced serum endotoxemia and improved insulin sensitivity in both animal models and human clinical trials [8, 20]. In the present study, supplementation with KGM decreased gut permeability, as well as increased the thickness of the mucus layer and gene expression level of tight junction proteins, indicating the beneficial effects of the bioactive dietary fiber on intestinal homeostasis in obese animals (Figure 2A−E). HFD or western diet reduced mucus thickness, increased gut permeability, and proinflammatory marker levels in mice, leading to an increase in serum LPS level and further inducing insulin resistance through toll‐like receptor‐4 (TLR‐4) in multiple tissues [21, 22, 23, 24]. Moreover, KGM not only decreased circulating LPS and proinflammatory factor levels but also insulin resistance, inferring that it may attenuate insulin resistance via the fortified gut barrier function (Figure 1J−K).

Diet is one of the most important factors that determine the composition of gut microbiota, which further influences gut health. HFD facilitated dysbiosis of gut microbiota, reducing the abundance of gut barrier–promoting microbes (such as *Lactobacillus* spp., *Bifidobacterium* spp., and *Akkermansia muciniphilia*) while augmenting the abundance of microbes implicated in impairing the intestinal barrier [25]. Supplementation with bioactive dietary fiber was reported to regulate gut microbiota and exert beneficial effects in various metabolic diseases. Herein, the abundance of *B. ovatus* was significantly increased by KGM intervention, and was negatively correlated with HOMA‐IR, as well as levels of serum endotoxin and TNF‐α (Figure 3F). A previous study evinced that the increased abundance of *B. ovatus* induced by Citrus polymethoxyflavones could reduce BCAA concentrations and alleviate insulin resistance in mice with metabolic syndrome [26]. Also, the colonization of *B. ovatus* promoted differentiation into goblet cells (related to the mucous layer of the intestine) and mitigated the inflammatory response in DSS‐induced colitic mice [27]. In our study, colonization of *B. ovatus* improved glucose homeostasis and insulin resistance, and decreased serum endotoxin levels, along with increasing the gene expression level of intestinal *Zo1*, *Ocln*, and *Muc2* in HFD‐induced obese mice (Figure 4). Altogether, these results suggested that *B. ovatus* may be a beneficial bacteria and is conducive to the alleviation of insulin resistance via enhancing intestinal barrier function.

The abundance of *B. ovatus* was significantly increased by KGM in both animal models and in vitro fermentation (Figure 3C,J). *B. ovatus* has been reported to utilize complex polysaccharides by activating different polysaccharide utilization loci (PULs) operons, such as *Bacova_03433*, *Bacova_03421*, and *Bacova_02092* [28]. KGM is a glucomannan hallmarked by β‐(1 → 4)‐linked d‐glucosyl and d‐mannosyl residues as the main chain with branches through β‐(1 → 6)‐glucosyl units [29]. Besides, *B. ovatus* is capable of degrading and growing on several complex plant cell wall polysaccharides, such as β‐mannan‐based dietary fibers. A putative β‐mannan PUL was previously identified in *B. ovatus* ATCC 8483 (*bacova_02087–02097*) and shown to be transcriptionally upregulated when KGM was introduced to the culture medium, among which two GH26 β‐mannanases (BoMan26A and BoMan26B) coded by bacova_02092–93, was considered important for KGM degradation [30, 31]. Nevertheless, the growth of *B. ovatus* was dramatically hampered after the deleption of gene *bacova_02087–02097* PUL [32]. We found that *B. ovatus* grows efficiently on a medium with KGM as the sole carbon source (Figure S2D–E), suggesting KGM as a critical dietary factor driving the growth of *B. ovatus*.

The gut microbiota influences host metabolism via modulation of metabolites, partly by mediating the interaction between the gastrointestinal system and other organs. Improvements in intestinal barrier function by bacteria‐derived metabolites have been widely reported [33]. The results that heat‐killed *B. ovatus* did not alleviate insulin resistance pointed out that metabolites produced by the bacteria may be critical to ameliorating insulin resistance, and IAA can be produced by *B. ovatus* (Figure 4D−F). IAA is a microbiota‐derived tryptophan metabolite that serves as an agonist of the AhR. AhR is widely expressed in the intestinal microenvironment and is activated by natural ligands to promote barrier integrity by upregulation of tight junction proteins in intestinal epithelial cell, and improve intestinal homeostasis through immune cells [3, 34]. For patients with metabolic syndrome and concomitant impaired AhR activity, supplementation with AhR ligands achieved by the administration of a *Lactobacillus* strain promoted metabolic homeostasis and improved obesity and insulin resistance [35]. The AhR agonist indigo also protects against obesity‐related insulin resistance through the modulation of gut homeostasis, especially by acting on the fortified gut barrier function [36]. Supplementation with IAA activated intestinal AhR (*Cyp1a1*, Figure 6L), accompanied by the increased gene expression of tight junction proteins and mucus layer thickness, led to the improvement of gut barrier function and amelioration of insulin resistance (Figure 5H−K). This finding sheds light on the underlying mechanisms driving *B. ovatus* proliferation by KGM to ameliorate insulin resistance via AhR activation.

## CONCLUSION

In the present study, our results indicated that gut microbiota plays a necessary role in regulating insulin resistance, and highlights the importance of gut barrier function during KGM intervention. *B. ovatus* is a primary degrader of KGM and promotes the production of IAA, leading to the regulation of intestinal barrier integrity by activation of intestinal AhR. This metabolic improvement could be exploited to selectively promote key members of the healthy microbiota using KGM‐based therapeutic interventions.

## METHODS

### Chemicals and reagents

High‐performance liquid chromatography‐grade acetonitrile and methanol were purchased from Merck (Darmstadt). LC‐MS grade formic acid was obtained from Sigma‐Aldrich. Authentic analytical reference standards for 5‐methoxytryptophan, 5‐hydroxytryptophan, tryptamine, 5‐methoxytryptamine, N‐acetyl‐5‐hydroxytryptamine, melatonin, indoleacetic acid, indole‐3‐propionic acid, indole‐3‐lactic acid, indole acrylic acid, indole‐3‐acetamide, 5‐hydroxyindole‐3‐acetic acid, and kynurenine were purchased from Aladdin. All aqueous solutions were prepared with ultrapure water produced by a Milli‐Q water purification system (Millipore).

### Preparation of KGM

Briefly, dry powder of konjac tubers was immersed in anhydrous ether and ethanol solution (45%, v/v) with constant stirring for 24 and 1.5 h to remove contaminants. The treated powder was mixed with 10 times distilled water with continuous stirring for 2 h followed by centrifugation at 4800 rpm/min, and collect the supernatant. The concentrated water extract was precipitated by adding four volumes of anhydrous ethanol at 4°C for 24 h to get crude polysaccharides. The KGM was purified according to our previous method [29, 37].

### Mice and treatments

All procedures for animal use were conducted in strict compliance with the National Research Council's Guide for the Care and approved by the Animal Care and Use Committee of Nanchang University [SYXK(GAN)2021‐0004]. All mice were housed under controlled conditions at 22 ± 2°C, 55 ± 10% relative humidity, and 12/12 h light/dark cycle with ad libitum access to food and water. All mice were randomly assigned to experimental groups, and the groups did not present differences in body weights before the treatments. To investgate the effect of KGM on mice during the development of insulin resistance, 8‐week‐old C57BL/6J specific‐pathogen‐free (SPF) mice were fed for 10 weeks with a standard chow diet or HFD (Research Diets, cat# D12492). Mice were supplemented daily with PBS or KGM (200 mg/kg) by gavage. To test the improvement effect of KGM on mice with insulin resistance, 6‐week‐old male mice were fed HFD for 8 weeks to induce insulin resistance and then treated with KGM for 4 weeks.

For FMT, fecal samples were collected daily from mice treated with glucomannan or PBS. Collected feces (100 mg) were resuspended in 1 mL of sterile anaerobic PBS, and centrifuged at 200*g* for 3 min at 4°C, and the supernatant was collected. After 1 week of Abx treatment, HFD‐fed mice were administered 100 μL of the above supernatant by gavage three times per week for 4 weeks.

To investigate the role of *B. ovatus* on the production of IAA, 8‐week‐old C57BL/6J SPF mice were treated with 2 × 10^8^ CFUs of *B. ovatus* in 200 μL of sterile anaerobic PBS by gavage twice per week and last for 1 week, feces were collected for the quantification of IAA.

To test the effects of *B. ovatus* on mice with insulin resistance, 8‐week‐old C57BL/6J SPF mice were fed for 10 weeks with HFD. Mice were treated with *B. ovatus*, heat‐killed *B. ovatus* or an equivalent volume of anaerobic PBS once per week.

To test the effects of IAA on mice with insulin resistance, 8‐week‐old C57BL/6J SPF mice were fed for 10 weeks with HFD. Mice were treated with control of 50 mg/kg IAA by oral gavage three times per week.

### Antibiotic treatment

Mice were treated with antibiotics in the drinking water for 1 week. The antibiotics consisted of vancomycin (0.5 mg/mL; Sigma), metronidazole (1 mg/mL; Sigma), kanamycin (1 mg/mL; Sigma), and ampicillin (1 mg/mL; Sigma). Antibiotic‐treated mice were maintained in sterile cages, given sterile food and water, and handled aseptically.

### Metabolic assays

OGTTs were performed after the mice fasted for 6 h. Blood glucose concentrations were measured with a glucometer from tail vein blood at 0, 15, 30, 60, and 90 min after oral administration of glucose (1.5 g/kg body weight). For ITTs, insulin (0.8 U/kg body weight) was administered via an intraperitoneal injection after 6 h of fasting.

### 16S rRNA gene sequencing and data analysis

Total DNA was extracted from colonic contents by QIAamp DNA Stool Mini Ki. The V3–V4 region of the bacterial 16S rRNA was amplified by polymerase chain reaction (PCR). Amplicons were extracted from 2% agarose gels and purified using the Common Agarose Gel DNA Recovery Kit (TIANGEN CO., LTD.). Purified PCR products were quantified by Qubit 3.0 (Life Invitrogen). Mix the DNA as recommended and the preparation of the DNA library included the repair ends and selected the library size, adenylate 3′ ends, ligate adapters, and enriched DNA fragments. Agilent 2100 Bioanalyzer System (Highly Sensitive DNA Chip) was used for the quality control of the library. The pooled DNA product was paired‐end sequenced on the Illumina MiSeq platform according to the standard protocols. After filtering the chimeras by USEARCH, sequences were clustered into Operational Taxonomic Units (OTUs) at a similarity cutoff value of R 97% using the UPARSE algorithm. A representative sequence of each OTU was assigned to the taxa at genus level in the optimized version of the RDP database (http://rdp.cme.msu.edu). Each unique OTU was subjected to BLAST against NCBI 16S database to identify closest match to the taxa at species level based on lowest *e*‐value and identity %. Abundances were recovered by mapping the de‐multiplexed reads to the UPARSE OTUs. A rarefied OUT table from the output files was further analyzed by a visualization toolkit. The resulting abundance table and taxonomic classification was loaded into R. Statistical analysis of differentially abundant sequences and taxa were performed by DESeq. 2 1.16.1 and the log_2_ fold changes (log_2_FC) represented the comparison against the reference level.

### Nontargeted metabolomics

For the nontargeted metabolomics analysis, feces (50 mg) were suspended in 50% MeOH (0.5 mL). Each sample was lysed using a tissue laser and subsequently vortexed for 20 min for metabolite extraction. All samples were subsequently centrifuged at 13,000*g* for 20 min at 4°C. The samples were detected using Shimadzu nexera X2 UPLC coupled to an AB Sciex Triple TOF 5600 mass spectrometer by following the method as described in our previous publication [38, 39]. The acquired row mass data were processed using commercial software Prognosis QI (Waters Corporation). A data matrix containing normalized ion intensities (variables), variable index (paired *m*/*z* targeted metabolomics retention time), sample names (observations), and isotope distribution was generated using *m*/*z* and retention time as the identifiers for each ion. The pooled QC samples were used for signal correction, and the resultant data matrices were further processed via the 80% rule. Metabolite annotation was made by searching MS and MS/MS information against the HMDB database (version: 4.0), and METLIN (version: 1.0.6499.51447). The mass error tolerance of ms1 and ms2 was set at 5 ppm.

### Targeted metabolomics

LC‐MS/MS system that composed of a Shimadzu Nexera X2 high‐performance liquid chromatography system (Kyoto) coupled to a Sciex 4500 triple quadrupole linear ion trap mass spectrometer (AB SCIEX) was used for the analysis of tryptophan metabolites. Chromatographic separation was employed with an ACQUITY UPLC CSH C18 column (2.1 × 100 mm, 1.7 μm; Waters), and mobile phase A contained 0.1% FA in water and mobile phase B 0.1% FA in acetonitrile. All analytes were detected in positive ion multiple reaction monitoring (MRM) mode. Chromatographic separation was performed using a linear gradient as follows: 0−4 min, 5%−40% B; 4−6 min, 40%−80% B; 6−8 min, 80% B; 8−10 min, 80%–5% B. Operational control of the LC‐MS/MS was performed with Analyst version 1.6.2, and quantitative analysis was performed using MultiQuant software (version 3.0.1).

Feces (10 mg) were suspended in 50% MeOH (0.3 mL) and lysed using a tissue laser. Samples were subsequently vortexed for 20 min for metabolite extraction. All samples were subsequently centrifuged at 13,000*g* for 20 min at 4°C. The supernatant was filtered through a membrane filter (pore size, 0.22 μm) for LC‐MS/MS analysis. For the quantification of IAA in culture supernatants of *B. ovatus*, culture supernatants were diluted with methanol (1:4, v/v), vortexed for 10 min, and centrifuged at 13,000*g* for 20 min for LC‐MS analysis.

### Gene expression analysis

Mouse tissues were frozen in liquid nitrogen and stored at −80°C. Standard phenol‐chloroform extraction was performed for the isolation of total RNA from frozen tissues with the TRIzol reagent. Complementary DNA was synthesized from 2 μg of total RNA with a Reverse Transcription Kit. The relative amount of each messenger RNA (mRNA) was calculated by normalizing to the β‐actin mRNA. Primer sequences for real time quantitative PCR (RT‐qPCR) are shown in Table S1.

### Bacterial strains and culture

For the in vitro fermentation, feces from mice were collected and cultured as described [40, 41]. Briefly, fecal samples were collected (keep them at 4°C) and used for inoculum preparation within 1 h after collection. The fecal pellet (100 mg) is resuspended in 1 mL sterilized PBS, then the samples were immediately homogenized using a vortex for 1 min. The fecal suspension was centrifuged to remove larger particles (550 g for 5 min at room temperature). The supernatant was harvested and 10 times diluted for incubation (5% inoculum level [v/v]) in medium (containing 5 g/L KGM as carbon source) under anaerobic conditions for 48 h.

For the bacteria isolation and identification, the fermented samples were serially diluted in sterile PBS, and spread on BHI agar plates. After incubation under anaerobic conditions at 37°C for 48 h, the colonies were picked, subcultured, and amplified by PCR. The single colonies were identified via 16S rRNA sequencing.

*B. ovatus* was cultured in 50 mL of GAM medium with at 37°C in an anaerobic chamber for 24 h. Cell pellets were obtained by centrifugation at 8000*g* for 10 min at 4°C. The cell suspension used for oral administration was prepared by suspending the cultured bacterial cells in oxygen‐free PBS to a final cell density of 1 × 10^9^ colony‐forming units (CFU)/mL. Heat‐killed *B. ovatus* was prepared by incubating an aliquot of *B. ovatus* suspension at 115°C for 30 min.

### Fecal SCFAs

SCFAs in feces were measured as described in our previous report [16]. Briefly, feces were diluted with deionized water at a ratio of 1:9. Diluted feces were vortexed and subjected to ultrasound for the extraction of SCFAs. Samples were placed in an ice bath and then centrifuged at 4800*g* for 20 min. The procedure was repeated and the supernatant was collected for the gas chromatography analysis.

### Serum biochemical analysis

Levels of serum lipids including total cholesterol, triacylglycerols, high‐density lipoprotein cholesterol (HDL‐c), and low‐density lipoprotein cholesterol (LDL‐c) were determined using an automatic biochemical analyzer (Mindray BS‐380). Nonesterified fatty acid (NEFA) concentration in the serum was determined using an assay kit (Nanjing Jiancheng Bioengineering Institute).

### Intestinal permeability

Intestinal permeability was assessed following oral administration of fluorescein‐isothiocyanate (FITC)‐dextran. Mice were fasted for 4 h then gavaged with FITC‐dextran (200 mg/kg body weight). After 90 min, 100 μL of blood was collected from the tip of the tail vein. The blood sample was kept in the dark and centrifuged at 3000*g* for 10 min to get a serum sample. Serum aliquots (20 μL) were plated in 96‐well plates and diluted to 200 μL with PBS, then measured at an excitation wavelength of 485 nm and an emission wavelength of 520 nm using a spectrophotofluorometer.

### Detection of serum endotoxin

Serum endotoxin was quantified using an endpoint chromogenic limulus amaebocyte lysate assay (Yeasen Biotechnology Co., Ltd.) according to the manufacturer's instructions.

### Statistical analysis

All statistical data were analyzed using SPSS version 26.0. All experimental data are reported as the means ± SEMs. The mouse samples were analyzed by two‐tailed Student's *t*‐test, Mann–Whitney *U* test, one‐way analysis of variance with Tukey's post hoc test or Dunnett's T3 post hoc test, Kruskal–Wallis test followed by Dunn's test. A *p* < 0.05 was considered to indicate significance.

## AUTHOR CONTRIBUTIONS

Shaoping Nie designed the study. Qixing Nie, Yonggan Sun, Chunhua Chen, and Qiongni Lin performed the experiments. Qixing Nie and Wenbing Hu interpreted the data and wrote the manuscript. All of the other authors revised and edited the manuscript. All authors read and approved the final manuscript.

## CONFLICT OF INTEREST STATEMENT

The authors declare no conflict of interest.

## ETHICS STATEMENT

The animal experiments (SYXK(GAN)2021‐0004) and ethics application (NCULAE‐20231103001) were approved by Science and Technology Department of JiangXi Province and the Animal Care and Use Committee of Nanchang University, respectively.

## Supporting information


**Figure S1**: KGM ameliorates HFD‐induced metabolic disorders.
**Figure S2**: KGM influenced the composition of gut microbiota.
**Figure S3**: Alleviation of insulin resistance by KGM is microbiota‐dependent.
**Figure S4**: *B. ovatus* ameliorates HFD‐induced metabolic disorders.
**Figure S5**: The changes of fecal SCFAs in different groups.

**Table S1**. PCR primers used in this study.

## Data Availability

The raw data of 16s rRNA sequencing is deposited in China National Microbiology Data Center (NMDC) with accession numbers NMDC10018595 (https://nmdc.cn/resource/genomics/project/detail/NMDC10018595). The code of microbiome profiling is available in Github (https://github.com/qixingnie/Script). Supplementary materials (figures, tables, scripts, graphical abstract, slides, videos, Chinese translated version, and updated materials) may be found in the online DOI or iMeta Science http://www.imeta.science/.
