# Supplementary material for: Glucomannan promotes Bacteroides ovatus to improve intestinal barrier function and ameliorate insulin resistance
Source: Imeta. 2024 Jan 3;3(1):e163. doi: 10.1002/imt2.163 (PMC10989147; doi:10.1002/imt2.163)

**Supporting information to:**

**Glucomannan promotes *Bacteroides ovatus* to improve intestinal barrier function and ameliorate insulin resistance**

**Running title: Glucomannan ameliorates insulin resistance**

Qixing Nie^1^, Yonggan Sun^1^, Wenbing Hu^2^, Chunhua Chen^1^, Qiongni Lin^1^, and Shaoping Nie^1,*^

^1^ State Key Laboratory of Food Science and Resources, China-Canada Joint Lab of Food Science and Technology, Key Laboratory of Bioactive Polysaccharides of Jiangxi Province, Nanchang University, Nanchang 330047, China

^2^ College of Grain Science and Technology, Jiangsu University of Science and Technology, Zhenjiang 212001, China

^*^ Correspondence: [spnie@ncu.edu.cn](mailto:spnie@ncu.edu.cn) (Shaoping Nie)

- **Supplementary Figures**

**Figure S1. KGM** **ameliorates HFD-induced metabolic disorders**

(A) Energy intake. (B) Serum TG. (C) Serum TC, HDL-c, and LDL-c. (D) Serum NEFA. (E and F) Serum ALT and AST levels in mice treated with KGM (related to Figure 1A). (G) Energy intake. (H) Serum TG. (I) Serum TC, HDL-c, and LDL-c. (J and K) Serum ALT and AST levels in obese mice treated with KGM (related to Figure 1L). **p* < 0.05; ** *p* < 0.01.


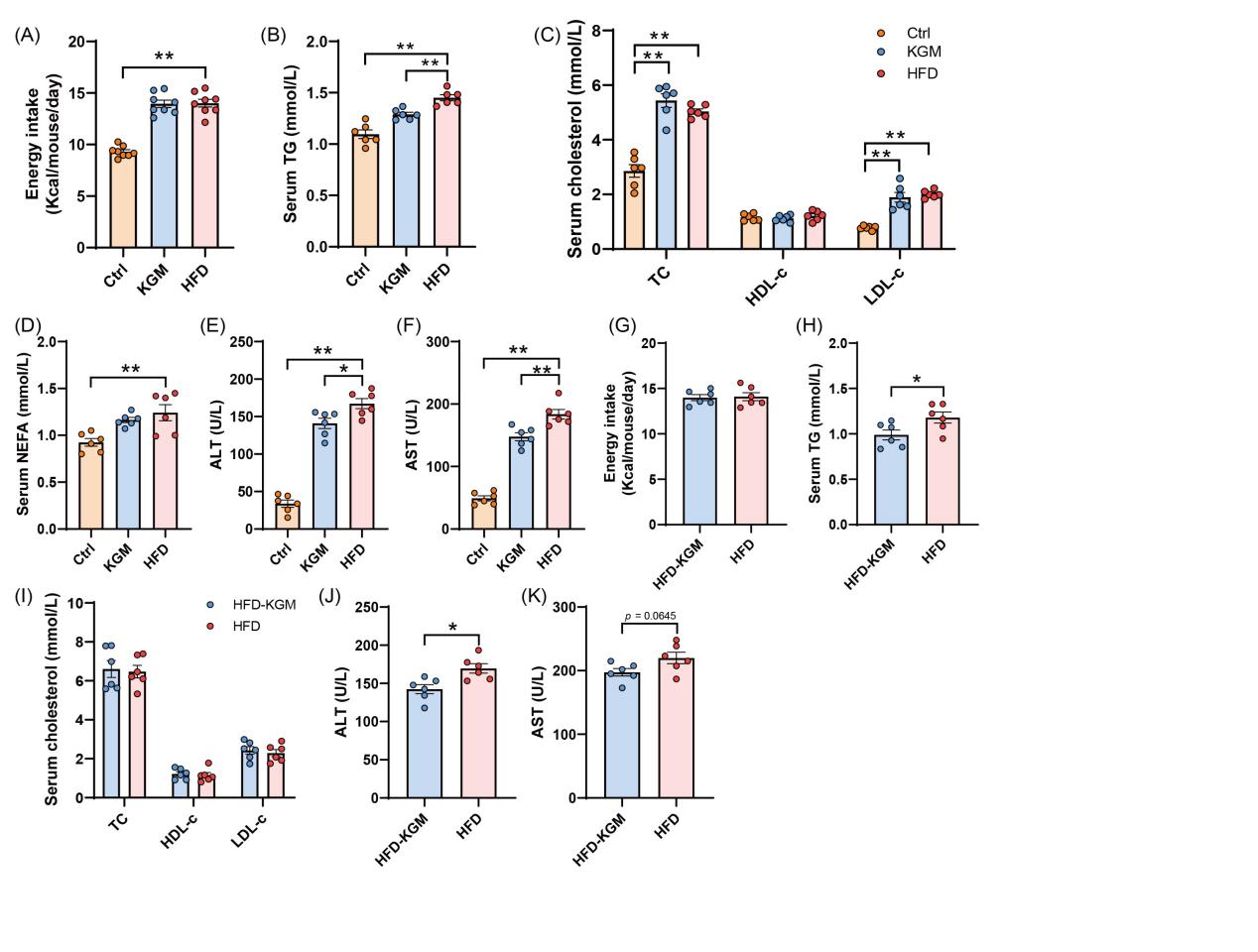


**Figure S2.** **KGM influenced the composition of gut microbiota**

(A) Relative abundance at phylum level after KGM intervention. (B) Relative abundance at species level after KGM intervention. (C) Comparison of relative abundance of bacteria in the fermented sample between 0 and 48 h at genus level in medium with KGM as a sole carbon source by LEfSe. (D) Growth curves of *B. ovatus* in basal medium. (E) Changes of total carbohydrates of basal medium during the growth of *B. ovatus.*


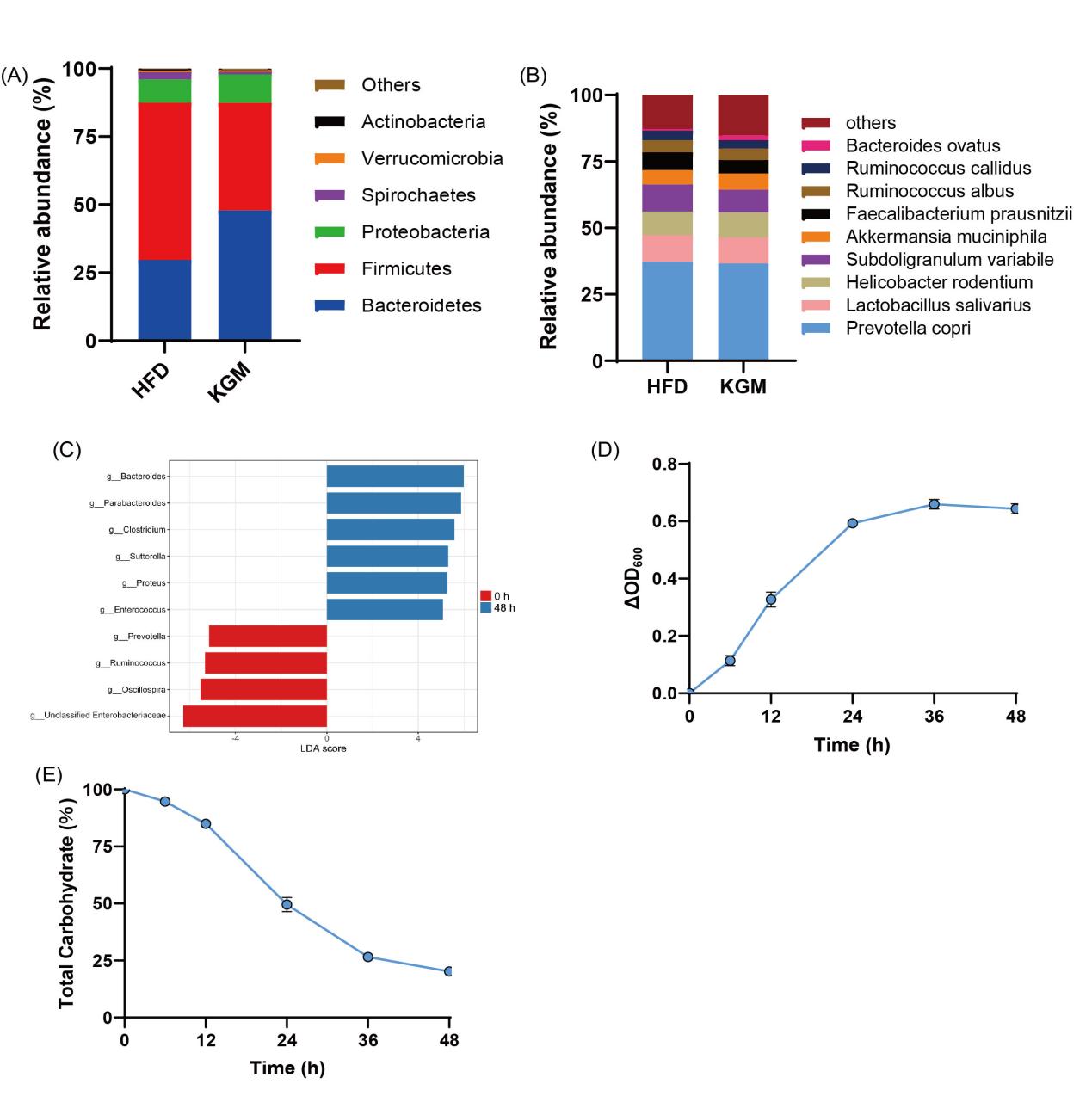


**Figure S3.** **Alleviation of insulin resistance by KGM is microbiota-dependent**

(A) Mice with insulin resistance were treated with antibiotic cocktails for 7 days to deplete gut microbiota, followed by KGM administration. Changes of (B) Body weight, (C) Energy intake, (D) OGTT, (E) OGTT associated area under the curve (AUC) values, (F) ITT, (G) Fasting insulin level, (H) HOMA-IR index in different groups. (I) Mice with insulin resistance were treated with antibiotic cocktails for 7 days to deplete gut microbiota, and fecal homogenates from KGM-treated or untreated obese mice were orally transmitted into antibiotic-treated recipient mice. Changes of (J) Body weight, (K) Energy intake, (L) OGTT, (M) OGTT associated area under the curve (AUC) values, (N) ITT, (O) Fasting insulin level, (P) HOMA-IR index after FMT. **p* < 0.05; ** *p* < 0.01.


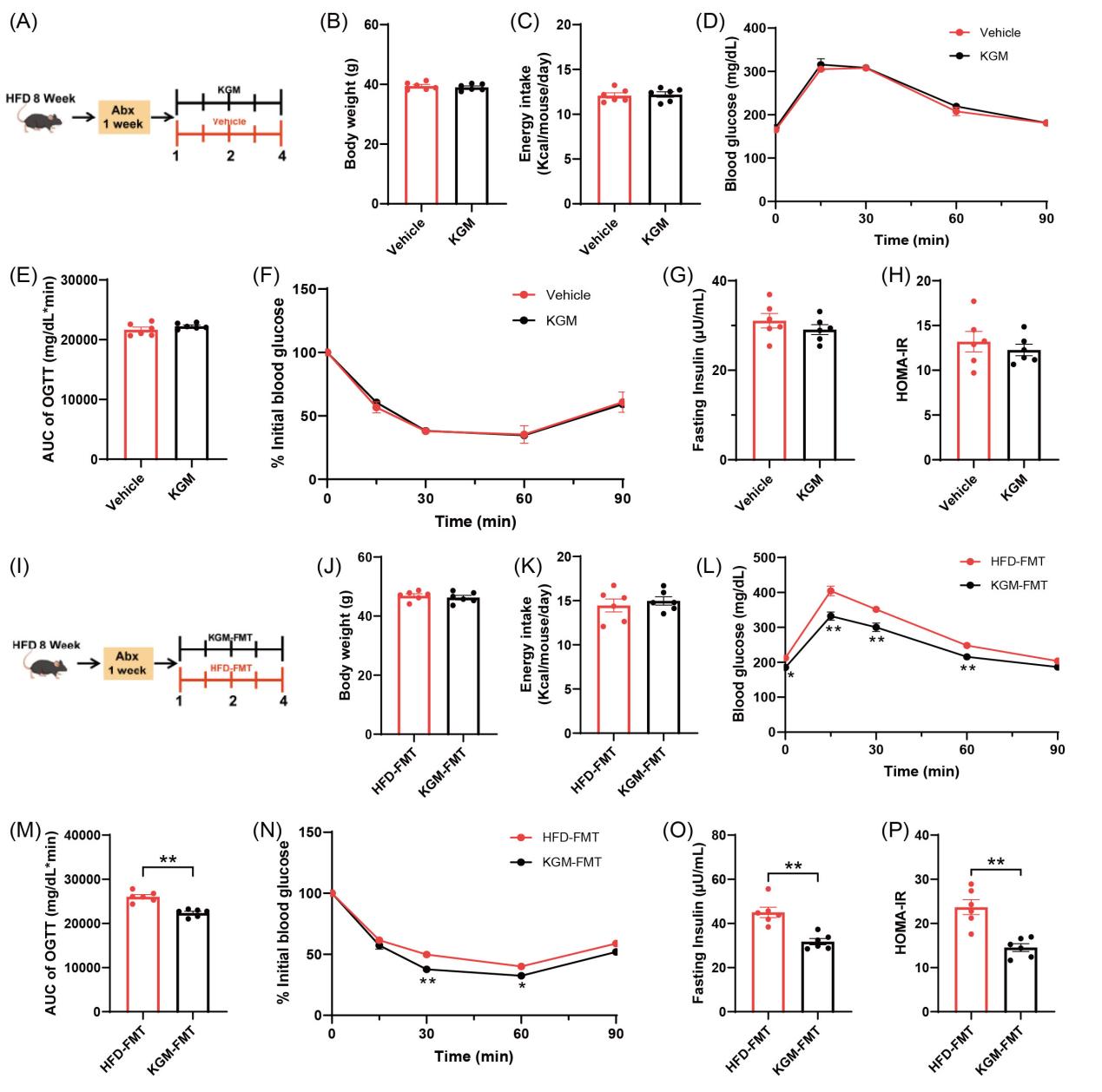


**Figure S4. *B.ovatus* ameliorates HFD-induced metabolic disorders**

(A) Energy intake. (B) Serum TG. (C) Serum TC, HDL-c, and LDL-c. (D) Serum NEFA. (E and F) Serum ALT and AST levels in mice treated with *B.ovatus*. **p* < 0.05; ** *p* < 0.01.


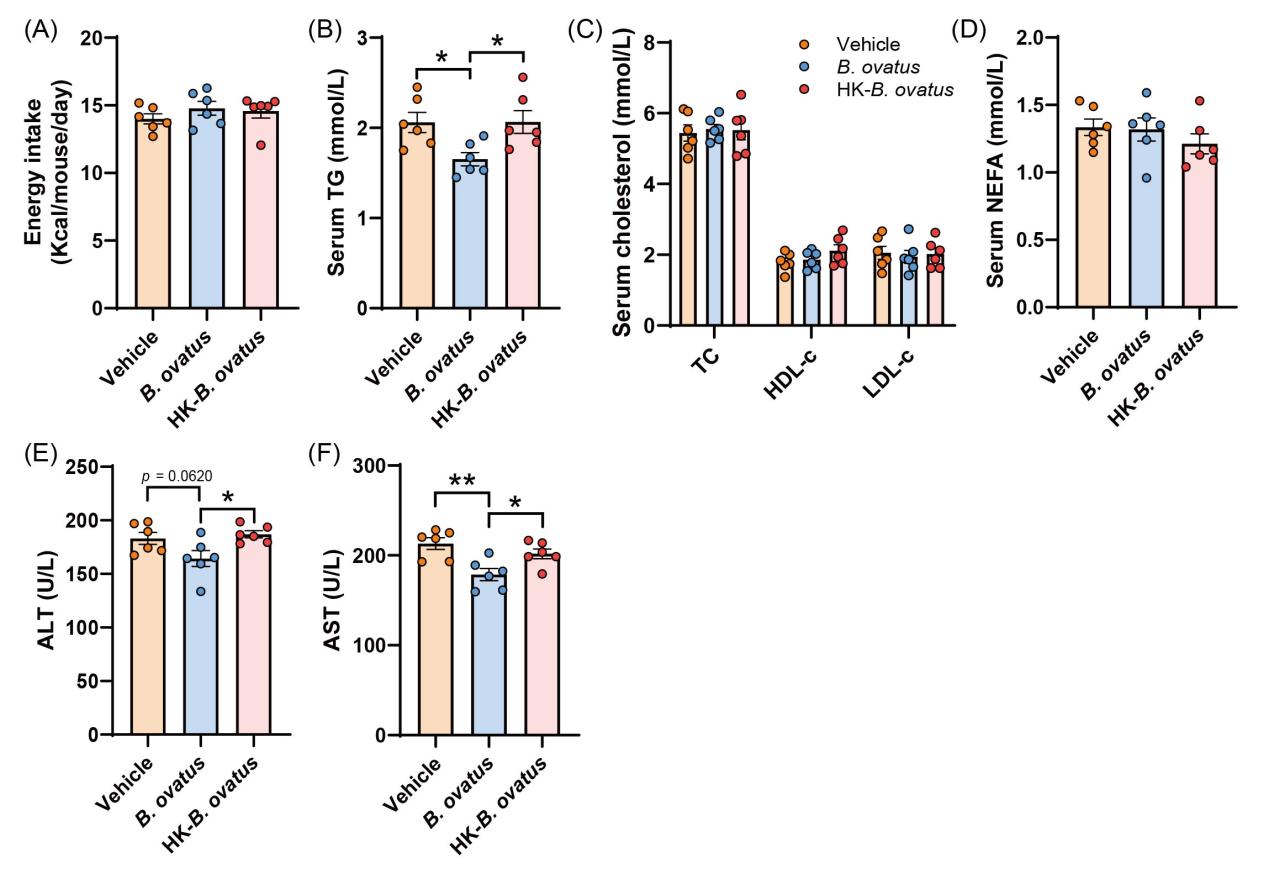


**Figure S5. The changes of fecal SCFAs in different groups**

(A) The intensity of IAA in fecal samples of mice after antibiotic treatment and FMT. (B-C) The relative expression of intestinal *Ahr*, *Cyp1a1*, *Reg3g*, *Il22* by *B. ovatus* and KGM treatment. (D-E) Change of fecal SCFAs level after *B. ovatus* and KGM treatment. **p* < 0.05; ** *p* < 0.01.


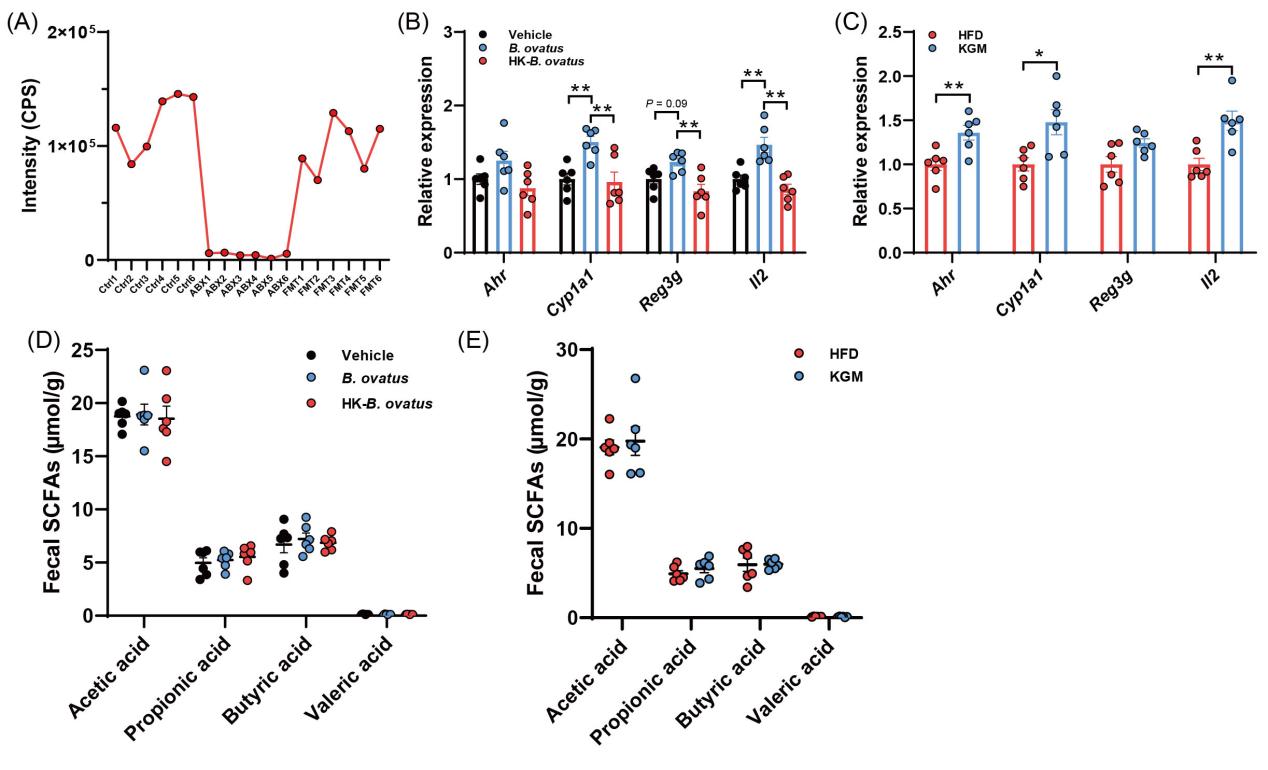

Supplement: Supplementary file 1 — Figure S1: KGM ameliorates HFD‐induced metabolic disorders. Figure S2: KGM influenced the composition of gut microbiota. Figure S3: Alleviation of insulin resistance by KGM is microbiota‐dependent. Figure S4: B. ovatus ameliorates HFD‐induced metabolic disorders. Figure S5: The changes of fecal SCFAs in different groups. [file IMT2-3-e163-s001.docx]
